# Supplementary material for: The Effect of Arabinoxylan and Wheat Bran Incorporation on Dough Rheology and Thermal Processing of Rotary-Moulded Biscuits
Source: Foods. 2021 Sep 30;10(10):2335. doi: 10.3390/foods10102335 (PMC8535077; doi:10.3390/foods10102335)
Supplement: Supplementary file 1 [file foods-10-02335-s001.zip › foods-1370654-supplementary.pdf]

Supplementary Material

Table S1: The chemical composition (% of total weight) of refined flour (RF), whole flour with large bran fraction (WFL), and whole flour with small bran fraction (WFS).

Flour components (% of total weight)	RF	WFL	WFS
Starch	70.6	58.5	59.2
Moisture	13.9	13.2	12.1
Proteins	10.0	11.4	11.5
Total fiber	3.6	13.0	13.2
Lipids	1.3	2.0	2.1
Ash	0.6	1.9	1.9
